# Hip geometry in hip fracture patients in Greenland occurring over a 7.7-year period

**DOI:** 10.1186/s13018-021-02482-7

**Published:** 2021-05-25

**Authors:** Inuuteq Fleischer, Mogens Laursen, Stig Andersen

**Affiliations:** 1 Department of Orthopaedic Surgery, Queen Ingrid’s Hospital, Nuuk, Greenland; 2 Arctic Health Research Centre, Department of Clinical Medicine, Aalborg University Hospital, 9000 Aalborg, Denmark; 3 Department of Orthopaedic Surgery, Aalborg University Hospital, 9000 Aalborg, Denmark; 4 Department of Geriatric & Internal Medicine, Aalborg University Hospital, 9000 Aalborg, Denmark

**Keywords:** Hip geometry, Hip fracture, Osteoporosis, Arctic population, Greenland

## Abstract

**Background:**

Hip geometry influences hip fracture risk. Hip fractures are common, and they are associated with pain, disability, premature death and marked costs on society. Osteoporotic fractures are frequent in Arctic populations and increase with advancing age in this society with a steep rise in life expectancy. Greenland Inuit is a distinct ethnic group, and data on hip geometry is missing. We thus aimed to describe hip geometry in 7.7 years of consecutive hip fracture patients in Greenland.

**Methods:**

We evaluated collodiaphysial angle, femoral neck length, the outer and inner diameter of the femur at 2 and 5 centimetres below the centre of the lesser trochanter and the cortical thickness from pelvic and hip radiographs in all patients operated in Greenland over 7.7 years. We included all 84 patients with one non-fractured hip visible for geometric analysis. Analyses were conducted in duplicate.

**Results:**

We found a collodiaphysial angle of 134.8/132.6^o^ in men/women (*p* = 0.06) and a femoral neck length of 38.0/33.9 mm in men/women (*p* = 0.001). Cortical thickness was affected by sex in the adjusted analysis (*p* < 0.001). Cortical thickness index at 5 cm below the centre of the lesser trochanter decreased with age (*p* = 0.026) and may be influenced by height (2 cm below the centre of the lesser trochanter, *p* = 0.053).

**Conclusion:**

Our findings differed from European data and suggest a delicate balance in hip geometry in Arctic populations. Ethnic peculiarities influence the structure of the hip and may influence fracture risk. A focus on hip geometry and risk factors for osteoporotic fractures in Arctic populations is warranted.

## Background

Fracture risk differs with ethnicity, and ethnic differences in hip fractures risk between South African Blacks and Caucasian Whites were attributed to differences in hip geometry [1, 2]. Similarly, ethnic disparities exist between Inuit and Caucasians in bone metabolism [3] and geometry of the femur before adulthood [4], while hip geometry in the adult population in Greenland remains unknown. Also, a marked transition in society has occurred in Greenland over the past half-century [5]. This transition may be accompanied by changes in proximal femoral geometry that raise the risk of hip fracture, as suggested by data from a Caucasian population [6].

Hip fractures are important because they are associated with pain, disability, premature death, and marked costs on society [7]. Furthermore, the occurrence of hip fractures accelerates with age, and they are the predominant fracture in the 8th decade of life [8]. Thus, the steep increase in life span among Arctic population calls for attention to factors related to hip fractures in the ageing Arctic populations.

Vitamin D levels may be influenced by Arctic habitat [9] and affect the risk of fractures in populations in Greenland. In addition, ethnic differences occur in bone mineral density (BMD) [10], but BMD did not differ between Inuit and Caucasians in Greenland when adjusting for differences in body weight [11]. Still, osteoporotic fractures were frequent among older women in Greenland [12, 13].

Inuit is a distinct ethnic group with an ethnic-specific body build [14, 15], and a difference in geometry of the femur was found among Inuit aged below 20 years [4]. This difference may carry through to hip geometry in older adults. Moreover, complying with hip geometry when performing hip fracture surgery is essential for the outcome [16], and knowledge of hip geometry in older adults is necessary.

Hip geometry can be measured on plain radiographs to support fracture risk assessment [17, 18]. Thus, cortical index differed between non-osteoporotic and osteoporotic patients [17], and further measurements of hip geometry from plain radiographs are available from hip fractures patients in a parallel population [19].

Our study aimed to describe hip geometry and cortical thickness parameters among consecutive hip fracture patients in Greenland based on the hypothesis that hip geometry is similar between Greenlanders and Caucasians.

## Methods

### Setting

The Arctic is the region above the Arctic Circle, which is the line that circles the globe at approximately 66°N. Most of Greenland is situated above this line. Greenland is the world’s largest island, and it is sparsely populated with people living along the vast coastline. The total population of around 55,000 is mainly Inuit. The Inuit people are one of the two main branches in the Arctic, from which the Inuit living in Alaska, Canada, and Greenland are divided. Greenland hosts one Orthopaedic Department at the national hospital in the capital Nuuk. All hip fracture patients are transferred to Nuuk for surgery.

### Radiographs

We retrieved plain radiographs of patients admitted to the Orthopaedic Department at Queen Ingrid’s Hospital in Nuuk, Greenland, for a hip fracture over 7.7 years. The study period was from January 1st 2007, through September 1st 2015. Radiographs were taken using a digital X-ray **(**Toshiba RADREX Digital Radiography System Model DRAD-3000E). Radiographs included in the analysis had to fulfil criteria set up before analysis of the radiographs. These criteria were one non-fractured hip that was visible for geometric analysis to at least 5 cm below the prominent tip of the lesser trochanter. Eighty-four patients fulfilled the criteria for inclusion in the study. The routine was to take radiographs of the pelvis and the fractured hip. The exclusion criteria were a missing radiograph of the non-fractured hip.

### Analysis of radiographs

**Fig. 1:**
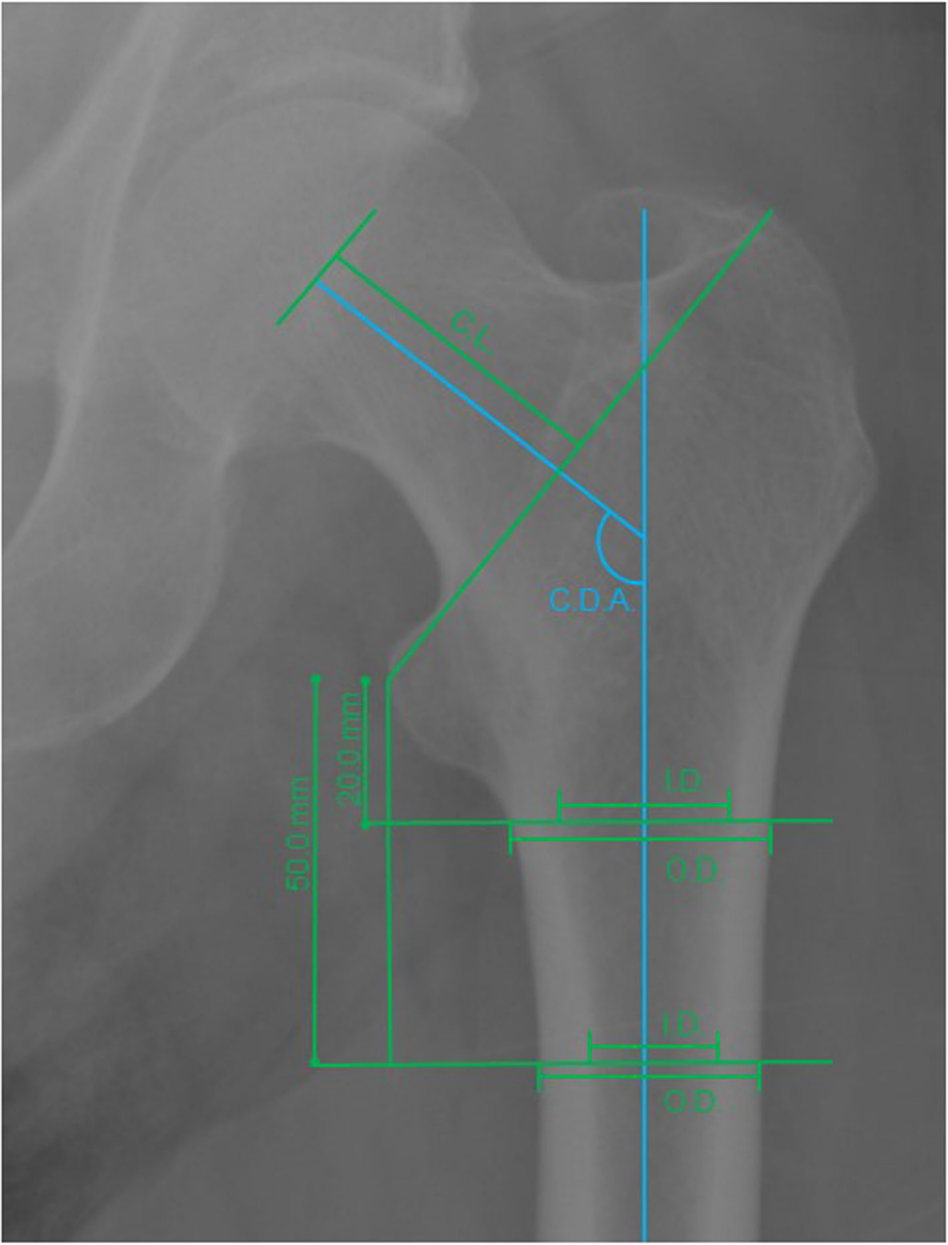
An illustration of measurements on the femur conducted on plain anteroposterior radiographs taken in hip fracture patients in Greenland. I.D.: inner diameter. O.D.: outer diameter. C.L.: collum length. C.D.A.: collodiaphysial angle

Radiographs were evaluated on a client review workstation (IMPAX 6.5. Solution). Measurements were conducted on plain anteroposterior radiographs of the pelvis, hip, or femur, as illustrated in Fig. 1. All measurements were performed in duplicate, and they were performed by a single evaluator (IF). The two measurements were done separately, at least 4 weeks apart. The second measurement was blinded to the results of the first measurement. Measurements were conducted as described and validated previously [18, 20] and detailed in Fig. 1.

Radiographic outcome parameters measured were collodiaphysial angle, femur neck length, outer, and inner femur diameter 2 and 5 cm below the lesser trochanter. Outcome parameters calculated were cortical thickness and cortical thickness index. Collodiaphysial angle was evaluated by first identifying the centre of the femoral head and drawing the line through this centre parallel to the femoral neck (Fig. 1). Second, the diaphyseal line was drawn through the centre of the femoral diaphysis. Third, the angle between these two lines was determined to depict the collodiaphysial angle (Fig. 1). The femoral neck length was determined by measuring the distance from the centre of the femoral head to the line drawn between the most prominent tip of the lesser and the upper tip of the major trochanter (Fig. 1). Finally, the outer and inner diameter of the femur was measured at 2 and 5 cm below the centre of the lesser trochanter (Fig. 1). We calculated the cortical thickness as the sub-periosteal width minus the endocortical width divided by two. The cortical thickness index was then calculated as the cortical thickness divided by the outer diameter. The cortical thickness index was calculated to account for differences in radiographic magnification and varying femoral size. Measurements were performed on the non-fractured femur at 2 and 5 cm below the most prominent tip of the lesser trochanter. Calibration was conducted individually for each radiograph.

### Demographics

The clinical parameters of sex, age, height and weight were retrieved from the hospital’s electronic medical records for each patient. The study cohort consisted of 56 (66.7%) women and 28 (33.3%) men with a mean age of 74 and 71. Details are provided in Table 1, along with height, weight and BMI.

**Table 1:** Descriptives of patients included in the study to describe hip geometry in Greenland hip fracture patients

	Men				Women				P ^a^
	Mean	SD	Median	25;75 percentiles	Mean	SD	Median	25;75 percentiles	
Age (years) ^b^	70.9	11.3	70.8	64.4;80.6	74.0	9.8	73.9	69.5;79.2	ns
Height (cm) ^c^	166.0	7.8	165.0	161.0;170.0	154.6	7.0	154.9	150.0;158.0	< 0.001
Weight (kg) ^d^	69.0	18.9	65.0	55.5;77.2	55.3	13.2	55.0	43.0;65.6	0.016
BMI (kg/m^2^) ^c^	24.7	5.5	23.9	21.9;27.6	23.1	4.4	23.0	19.5;25.0	ns

^a^T-test

^b^Number of patients was 21 men and 16 women

^c^20 missing

^d^19 missing

Data retrieval and analysis were conducted after approval by the Ethics Committee for Scientific Research in Greenland, and individual consent was not required (ethics committee reference no. 2013-16).

### Statistical analysis

Frequencies are given in both mean and standard deviation (SD) and median with 25 and 75 percentiles for descriptives of the participants. These were compared using the t test as all variables followed the normal distribution as tested using the Kolmogorov-Smirnov test. The data were further tested in linear regression analysis after checking for linearity, homogeneity of variance and distribution of data. BMI was not included in the regression analysis due to collinearity with parameters of body proportions. The variance inflation factor did not exceed tolerance for any of the remaining variables. Dependent variables entered were collum length, collodiaphysial angle, cortical thickness and cortical thickness index at 2 and 5 cm below the tip of the lesser trochanter. Explanatory variables entered were age, sex, height and weight. Regression analysis was performed first as a univariate analysis. Subsequently, multivariate analysis was done except for collodiaphysial angle as none of the variables in the univariate analysis influenced the dependent variables. All analyses were performed using the Statistical Package for the Social Sciences version 13.0 (SPSS Inc., Chicago, IL). A p value of less than 0.05 was considered significant.

## Results

Characteristics of the Greenlandic patients included in the study are provided in Table 1.

Table 2 gives the measured and calculated geometric data for the proximal femur. The gender difference in collodiaphysial angle (mean, 134.8/132.6° in men/women) was limited in the direct comparison (Table 2). Femur neck length differed with sex (*p* = 0.001), as shown in Table 2. Outer diameter differed with sex at 2 and 5 cm below the prominent tip of the lesser trochanter (both, *p* < 0.001), while the inner diameter differed with sex at only 2 cm (*p* = 0.028). The cortical thickness showed distinct gender differences at 2 and 5 cm below the tip of the lesser trochanter (both, *p* < 0.001). In comparison, the cortical thickness index differed most markedly at 5 cm below the lesser trochanter (*p* = 0.003).

**Table 2:** Measures of hip geometry among patients operated for hip fracture at Queen Ingrids Hospital in Nuuk over a 7.7-year period

	Men				Women				P ^a^
	Mean	SD	Median	25;75 percentiles	Mean	SD	Median	25;75 percentiles	
Collodiaphysial angle (degree)	134.8	5.0	134.6	130.9;139.0	132.6	4.7	132.7	129.9;135.3	0.057
Femur neck length (mm)	38.0	5.5	38.9	33.2;42.7	33.9	5.0	34.3	29.8;37.1	0.001
Outer femur diameter (mm)									
2 cm below lesser trochanter	32.6	3.2	32.4	30.1;34.9	29.0	2.5	29.0	26.8;30.7	< 0.001
5 cm below lesser trochanter	29.2	3.0	29.4	27.0;32.0	26.3	2.6	26.1	24.1;28.3	< 0.001
Inner femur diameter (mm)									
2 cm below lesser trochanter	21.9	3.3	21.3	19.6;23.8	20.4	2.7	20.7	18.6;22.3	0.028
5 cm below lesser trochanter	15.7	2.6	14.8	13.7;17.8	15.7	2.7	15.4	13.7;17.0	ns
Cortical thickness (mm) ^b^									
2 cm below lesser trochanter	5.4	1.0	5.4	4.6;6.0	4.3	0.9	4.2	3.5;4.9	< 0.001
5 cm below lesser trochanter	6.7	1.3	6.6	5.8;7.5	5.3	1.1	5.2	4.4;6.0	< 0.001
Cortical thickness index ^c^									
2 cm below lesser trochanter	0.16	0.03	0.17	0.15;0.19	0.15	0.03	0.14	0.13;0.17	0.022
5 cm below lesser trochanter	0.23	0.03	0.25	0.20;0.26	0.20	0.04	0.20	0.18;0.23	0.003

^a^ T-test

^b^ Cortical thickness calculated as (outer diameter–inner diameter)/2

^c^ Cortical thickness index calculated as ((outer diameter–inner diameter)/2)/outer diameter

**Fig. 2:**
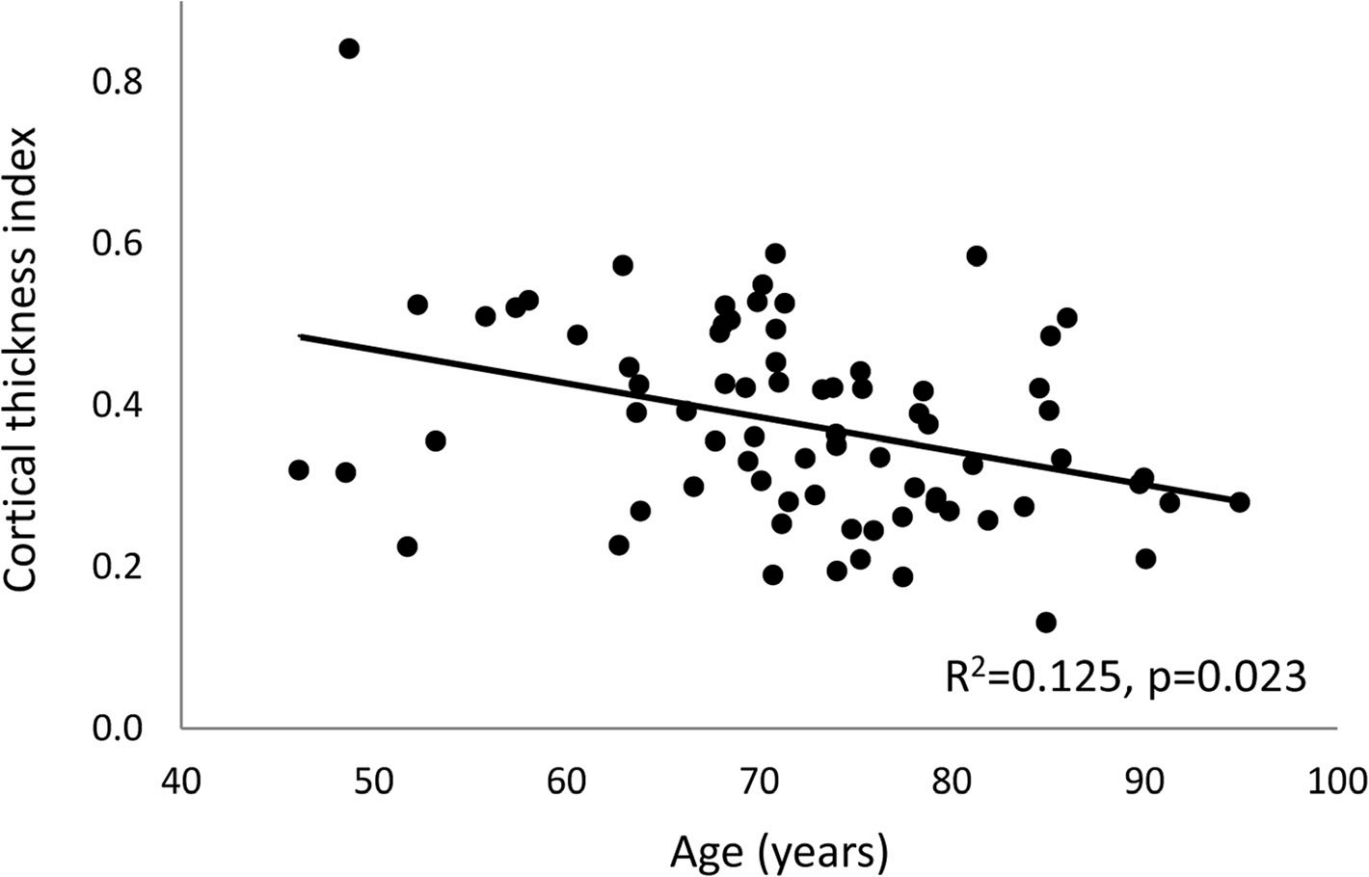
The age related decrease in cortical thickness index in hip fracture patients in Greenland over 7.7 years

Table 3 lists the univariate and multivariate analysis of factors important to collodiaphysial angle, collum length, cortical thickness and cortical thickness index. Collodiaphysial angle differed between men and women in the adjusted analysis (Table 3). Collum length was influenced by height, weight, sex and age in the adjusted analyses. Cortical thickness was influenced by age, sex, height and weight at both 2 cm and 5 cm below the lesser trochanter in the unadjusted analysis, while sex was the dominant factor in the adjusted analysis. Cortical thickness was affected by age, sex and height at both sites in the unadjusted analysis while only by the height at 2 cm in the adjusted analysis and only by age at 5 cm below the lesser trochanter. This decrease is illustrated in Fig. 2 (adjusted comparison, *p* = 0.026).

**Table 3:** Linear regression analysis of factors important to variables that are descriptive of the hip among patients operated for hip fracture in Greenland

	Univariate analysis								Multivariate analysis							
	Age		Sex		Height		Weight		Age		Sex		Height		Weight	
	Beta	*P*-value	Beta	*P*-value	Beta	*P*-value	Beta	*P*-value	Beta	*P*-value	Beta	*P*-value	Beta	*P*-value	Beta	*P*-value
Collodiaphysial angle	-0.05	ns	0.21	0.057	-0.15	ns	-0.07	ns	-0.18	ns	0.44	0.033	-0.38	0.066	0.17	ns
Collum length	-0.26	0.016	0.35	0.001	0.37	0.034	-0.07	ns	-0.30	0.048	0.36	0.033	0.43	0.044	-0.43	0.031
Cortical thickness																
2 cm below	-0.22	0.047	0.47	<0.001	0.51	0.003	0.39	0.023	-0.08	ns	0.59	<0.001	0.29	ns	0.16	ns
5 cm below	-0.29	0.010	0.50	<0.001	0.52	0.003	0.34	0.053	-0.19	ns	0.45	0.013	0.30	0.093	0.10	ns
Cortical index																
2 cm below	-0.21	0.054	0.25	0.022	0.35	0.053	0.23	ns	-0.22	ns	-0.04	ns	0.35	0.053	0.11	ns
5 cm below	-0.35	0.002	0.33	0.004	0.36	0.054	0.23	ns	-0.41	0.026	0.15	ns	0.12	ns	0.28	ns

The results of the repeated measurements of the radiographs did not show any systematic error in outer diameter or inner diameter, collum length, collodiaphysial angle, CT or CI (all, ns).

## Discussion

Our evaluation of hip geometry among hip fracture patients in Greenland identified an influence on hip geometry by height, sex and age while an influence of weight was limited. The impact of age on the cortical index was marked, as was the influence of sex and height on cortical thickness. Height had a major impact on collum length, and, interestingly, age also influenced collum length. These findings differ from those of parallel Scandinavian populations and suggest that hip geometry differs between Greenlanders and Caucasians.

Measurements of hip geometry can be performed on plain radiographs [17]. Such data may provide useful information to support fracture risk assessment [18] and osteoporosis evaluated from bone mineral density measurements [17]. Sah and colleagues reported cortical index measured on plain radiographs in patients with and without osteoporosis diagnosed by DXA in the same patients [17]. They found a marked difference in cortical index between non-osteoporotic and osteoporotic patients (0.55 vs 0.46) at 3 cm below the lesser trochanter [17]. This finding supports the impact of the cortical index on the risk of osteoporosis, and other measures of the cortical structure may be relevant to include to strengthen the estimate.

Beck reported data on hip geometry from NHANES III [21]. The cortical index was 0.480 in men and 0.377 in women at 2 cm below the lesser trochanter in their data and markedly lower in our data, 0.160 and 0.150, respectively. Furthermore, Beck and colleagues reported subperiosteal diameter of 3.62 and 3.20 cm in men and women, respectively, similar to both the 3.15 in Caucasian women reported by Nelson [22] and to a parallel group of hip fracture patients at our hospital in Denmark. In contrast, we found lower values in Greenland of 3.26 and 2.90 cm in men and women, respectively. The values reported from NHANES III and Nelson were based on the general population [21, 22], while our data are from patients with a fracture at the hip. A lower femoral neck cortical thickness in hip fracture patients than in individuals without fracture is in keeping with previous reports on the influence of ethnicity on hip geometry and fracture risk [23]. Thus, our data support the notion that cortical thickness may support hip fracture risk assessment.

Koeppen and colleagues reported cortical index values among patients with femoral subtrochanteric or shaft fractures [19]. Their population is parallel to our patients in Greenland except for Swedish origin. The cortical index was 0.37 at 5 cm below the lesser trochanter, and it was thus 0.14 higher than our patient’s cortical index of 0.23. The cortical index was lower in a parallel group of hip fracture patients at our hospital in Denmark and other Caucasians with a hip fracture than in the general Caucasian population [19, 21]. Still, it was lowest in Inuit with a hip fracture. This lower cortical index among hip fracture patients in Greenland may suggest that more parameters are at play for bone strength and fracture risk.

Nelson reported the outer/inner diameter of the femoral shaft at 2 cm below the lesser trochanter to be 3.15/2.05 cm in White and 3.14/1.99 cm in Black women [22]. However, we found diameters of 2.90/2.04 cm. Thus, the cortical thickness was 0.43 cm in the patients from Greenland compared to 0.55 cm in White and 0.57 cm in Black Americans in the study by Nelson [22], which is comparable to hip fracture patients at our hospital in Denmark. The markedly lower cortical thickness in our findings from Greenland further supports the notion that other parameters may influence hip fracture risk among patients in Greenland.

The collodiaphysial angle was reported previously to be between 124 and 129° [1]. However, this angle was 133° in our study. Such a higher angle suggests a lower risk of hip fracture in Greenlanders than Whites and Blacks and may support the assessment of femoral fracture risk.

Femoral neck length has been reported to be between 4.3 and 4.7 cm in non-Inuit women [1], and it was 3.25 cm around 1910, growing to 3.50 cm in the 1980s in Caucasian Scots [4]. Geometric changes were reported to occur over time, but it was estimated that ethnicity was the primary determinant of femoral size and shape [24]. Ethnic differences in femoral neck length contributed to differences in hip fracture risk [25]. We found a femoral neck length in women in Greenland to be 3.39 cm. This shorter femoral neck is more than can be explained by the difference in height between Caucasian and Inuit populations [5, 15]. Our finding thus suggests a shorter femoral neck in Greenland, and this could influence hip fracture risk.

Compared to Caucasians [5, 15], the higher BMI in Inuit could lower the hip fracture risk as weight influenced both geometric strength and hip fracture risk [26]. Therefore, this is an additional factor to include when estimating the influence of ethnicity on fracture risk.

Greenland is an Arctic environment with limited sun exposure and heavy clothing, and low vitamin D levels may be expected. However, the traditional Greenlandic diet is rich in vitamin D, and populations 400 km north of the Arctic Circle were not vitamin D deficient [27, 28]. Still, the powerful hormonal mechanisms that regulate calcium metabolism differ between Native and European populations [3], and ethnic differences are likely to apply to other aspects of skeletal health, including bone structure and strength.

Life expectancy in Greenland is around 70 years, but it is rising steeply [29]. This rising life expectancy influences the risk of osteoporosis as vertebral fractures are present and frequent [12, 13, 30]. Hip fractures occur one decade later in life than vertebral fractures, and a steep rise in the occurrence of hip fractures may be expected based on the ageing population in the Arctic.

Moreover, osteosarcopenia, as assessed by muscle volume, is an additional contributor to the risk of hip fracture [31] and may be considered in the growing elderly population in Greenland. Thus, our findings support careful monitoring of hip fracture occurrence and the development of a fracture risk assessment method applicable to Arctic populations. The vast geography and logistical constraints in the Arctic necessitate the use of local technology, and analysis of femoral geometry from hip radiographs may provide an opportunity to raise health care service in rural areas. An investigation of differences between Greenland’s population and other Arctic people is warranted. There is a need to establish specific outcome parameters and then analyse the influence of factors such as age, sex, height, weight and geometric parameters of the hip have on the fracture risk. These parameters may support fracture risk assessment in Arctic populations.

The number of patients included in our study was limited due to the population size of Greenland and the lack of available data on hip fracture patients before 2007. Furthermore, the low number of hip fracture patients is in keeping with a low frequency of hip fractures seen in a population with a median life expectancy of 70 years. However, we included all hip fracture patients operated in Greenland over 7.7 years. There is only one hospital with expertise in orthopaedic surgery, and hip fracture patients from all of Greenland are transported to the referral hospital in Nuuk for surgery. This logistic challenge and economic burden on health care will increase with the predicted rise in hip fracture frequency.

A further limitation to our study is the lack of a direct comparison with a Caucasian control group. However, our findings among hip fracture patients are compared to findings by others in similar patients and a group of hip fracture patients at our hospital in Denmark. Thus, our results provide some insight into the risk of hip fracture among Arctic populations and should encourage further data on this topic.

In conclusion, we found a higher collodiaphysial angle and shorter femoral neck than in parallel Scandinavian populations suggesting a lower hip fracture risk among people in Greenland. Conversely, we also found a smaller outer diameter with a similar inner diameter and a lower cortical index than the parallel Scandinavian populations, suggesting a higher hip fracture risk in people in Greenland. We thus found distinct differences between populations in Greenland and Scandinavia that conform to the notion that hip geometry is not similar between Greenlanders and Caucasians. Our findings have opposite effects on fracture risk, and they suggest a delicate balance. This balance may change with lifestyle changes and increase the risk of hip fracture, which rises in the ageing Arctic people. Finally, ethnic peculiarities are likely and may influence fracture risk prediction. Hence, a focus on hip geometry, bone metabolism and risk factors for osteoporotic fractures in Arctic populations is warranted. Future studies across Arctic populations may contribute to a composite score to support fracture risk assessment.

## Data Availability

The study is conducted in a small population, which restricts the availability of data. However, data extracts may be shared by contact with the corresponding author.
